# Plant beta-turnover rather than nestedness shapes overall taxonomic and phylogenetic beta-diversity triggered by favorable spatial–environmental conditions in large-scale Chinese grasslands

**DOI:** 10.3389/fpls.2024.1285787

**Published:** 2024-05-28

**Authors:** Zhenyu Yao, Yue Xin, Zhaoxia Ma, Liqing Zhao, Wenkui Mu, Jianying Guo, Arshad Ali

**Affiliations:** ^1^Yinshanbeilu Grassland Eco-hydrology National Observation and Research Station, China Institute of Water Resources and Hydropower Research, Beijing, China; ^2^Institute of Water Resources for Pastoral Areas Ministry of Water Resources, Hohhot, China; ^3^Inner Mongolia Key Laboratory of Grassland Ecology and School of Ecology and Environment, Inner Mongolia University, Hohhot, China; ^4^Beijiao Park, Hohhot, China; ^5^Inner Mongolia Hohhot Meteorological Bureau, Hohhot, China; ^6^Forest Ecology Research Group, College of Life Sciences, Hebei University, Baoding, Hebei, China

**Keywords:** biodiversity, climate, Leymus chinensis communities, soil, topography

## Abstract

**Introduction:**

Although it is widely acknowledged that biodiversity maintains plant community assembly processes, exploring the patterns and drivers of beta-diversity (*β*-diversity; species variation among local plant communities) has received much less attention compared to alpha-diversity (*α*-diversity; species variation within a local plant community). Here, we aim to examine the patterns and spatial–environmental drivers of taxonomic and phylogenetic *β*-diversity, and their components such as species turnover and nestedness, in large-scale *Leymus chinensis* grassland communities.

**Methods:**

We collected plant community data from 166 sites across widely distributed *L. chinensis* communities in northern China, and then calculated the taxonomic and phylogenetic *β*-diversity indices (overall, turnover and nestedness) using a pairwise dissimilarity approach. To assess the effects and to explain the variation in the patterns of *β*-diversity, we collected data on geospatial, climate and soil conditions. We applied descriptive statistics, Mental correlations, and multiple linear regression models to assess the patterns and spatial–environmental drivers of *β*-diversity.

**Results:**

The *β*-turnover, as compared to *β*-nestedness, exhibited a predominant influence, constituting 92.6% of the taxonomic *β*-diversity and 80.4% of the phylogenetic *β*-diversity. Most of the spatial–environmental variables were significantly positively correlated with the overall taxonomic and phylogenetic *β*-diversity and *β*-turnover, but not with *β*-nestedness. Climatic factors such as MAP and MAT were the strongest predictors of both taxonomic and phylogenetic *β*-diversity and *β*-turnover. The variance partitioning analysis showed that the combined effects of spatial and environmental factors accounted for 19% and 16% of the variation in the taxonomic and phylogenetic *β*-diversity (overall), 17% and 12% of the variation in the *β*-turnover, and 7% and 1% of the variation in the *β*-nestedness, respectively, which were higher than independent effects of either spatial or environmental factors.

**Discussion:**

At larger spatial scales, the turnover component of *β*-diversity may be associated with the species complementarity effect, but dominant or functionally important species can vary among communities due to the species selection effect. By incorporating *β*-diversity into grassland management strategies, we can enhance the provision of vital ecosystem services that bolster human welfare, serving as a resilient barrier against the adverse effects of climate change at regional and global scales.

## Introduction

A growing body of global evidence shows that biodiversity plays an important role in maintaining plant community assembly processes through species functional strategies which in turn regulate ecosystem functions such as biomass production, thereby providing numerous ecosystem services that underpin human well-being (van der Plas, 2019; Ali, 2023). Considering the multidimensional nature of biodiversity in maintaining the plant community assembly processes, biodiversity can be quantified as alpha-diversity (*α*-diversity; species variation within a local plant community) and beta-diversity (*β*-diversity; species variation among local plant communities) which can be further explained by changes in taxonomic, functional and phylogenetic diversity (Mori et al., 2018; Nakamura et al., 2020). Yet, exploring the patterns and drivers of *β*-diversity has received much less attention compared to *α*-diversity under diverse concepts, theories and analyses (Mori et al., 2018; van der Plas et al., 2023). Thus, further studies are needed to explore the patterns of plant *β*-diversity and its association with spatial–environmental (including climate and soil) factors which could enhance our understanding of plant community assembly processes, ecosystem functioning and biodiversity conservation.

In plant ecosystems, *β*-diversity considers variations in species identities and abundances among communities at a regional scale through species turnover (or substitution) and nestedness (differences in richness) (Baselga, 2010; Mori et al., 2018; Fu et al., 2019). In this context, species turnover refers to differences in species composition between communities caused by the phenomenon of species replacement, whereas species nestedness refers to changes due to an increase or decrease in species richness along an environmental gradient (Lennon et al., 2001; Staniczenko et al., 2013; Fu et al., 2019). Previous studies have revealed that potential mechanisms driving species turnover involve geospatial heterogeneity, environmental filtering and species competition (Cardoso et al., 2014; Boschilia et al., 2016; van der Plas et al., 2023). However, the mechanisms governing species richness differences encompass ecological niche diversity and the ecological processes of forming nested patterns (Legendre, 2014). In different communities, the overall composition of *β*-diversity components often varies, and their relative importance in community construction also depends on spatial scale (Boschilia et al., 2016; Bertuzzi et al., 2019). Consequently, decomposing *β*-diversity enables the discernment of spatial distribution patterns among different components, thereby elucidating the formation mechanisms and ecological significance of each component and facilitating an understanding of evolutionary processes (Baselga, 2010; Legendre, 2014).

Many previous studies have focused only on the level of taxonomic *β*-diversity in plant communities, which solely provides information regarding species differences among sites without considering the evolutionary aspects within a community (Graham and Fine, 2008; Qian et al., 2013). However, research indicates that incorporating phylogenetic information into *β*-diversity analysis provides significant assistance in exploring ecological processes such as plant community assembly processes and evolutionary patterns (Mori et al., 2018; Zheng et al., 2019; Ali, 2023). Therefore, conducting a concurrent analysis of both taxonomic and phylogenetic *β*-diversity holds paramount importance in obtaining a comprehensive understanding of the community assembly processes and dynamics within a plant ecosystem. Furthermore, numerous studies have indicated that patterns of *β*-diversity formation and maintenance are not exclusively driven by a single ecological process. Instead, their formation is influenced by the combined impacts of environmental filtering and dispersal limitation, exhibiting variations across different community types and spatial scales (Bellier et al., 2014; Soininen et al., 2018; Menegat et al., 2019). For instance, in studies of *β*-diversity within liana communities, it has been observed that community composition is jointly influenced by environmental filtering and dispersal limitation, with environmental filtering predominantly exerting control over the underlying ecological processes (Myers et al., 2013; Zhang et al., 2020). In contrast, several studies in grasslands have found dispersal limitation to be a major factor affecting taxonomic *β*-diversity (Pinto et al., 2014; Li et al., 2021). Hence, the driving mechanisms of *β*-diversity exhibit variations based on community type, climatic context, and scale (Mori et al., 2018; Gan et al., 2019; van der Plas et al., 2023).

Here, we broadly aim to highlight the crucial role of *β*-diversity in maintaining grassland biodiversity, community assembly processes and ecosystem functions. The specific objective of this study is to examine the patterns and spatial–environmental drivers of taxonomic and phylogenetic *β*-diversity in large-scale *L. chinensis* communities across Chinese grasslands. We anticipate that derived results from our study will offer valuable insights into the conservation and management of large-scale Chinese grasslands. The *Leymus chinensis* community is a unique vegetation type with a continuous distribution in the Eurasian steppe zone which is predominantly distributed in regions such as the Loess Plateau, Mongolian Plateau, and Daxinganling Mountains (Yao et al., 2022). This community is primarily observed in small and relatively moist habitats. Prior studies on *β*-diversity have predominantly concentrated on either local scales or diverse community types on a larger spatial scale (Chi et al., 2014; Qin et al., 2019; Hu et al., 2022), but fewer studies examining the same community type in a large-scale continuous distribution in a region. To this end, we address the following research questions: (1) What are the patterns and contributions of *β*-turnover and *β*-nestedness to each taxonomic and phylogenetic *β*-diversity (overall)? (2) How do spatial and environmental factors affect the patterns of taxonomic and phylogenetic *β*-diversity and their components, and what are their relative importance?

## Materials and methods

### Study area and field investigation

**Figure 1:**
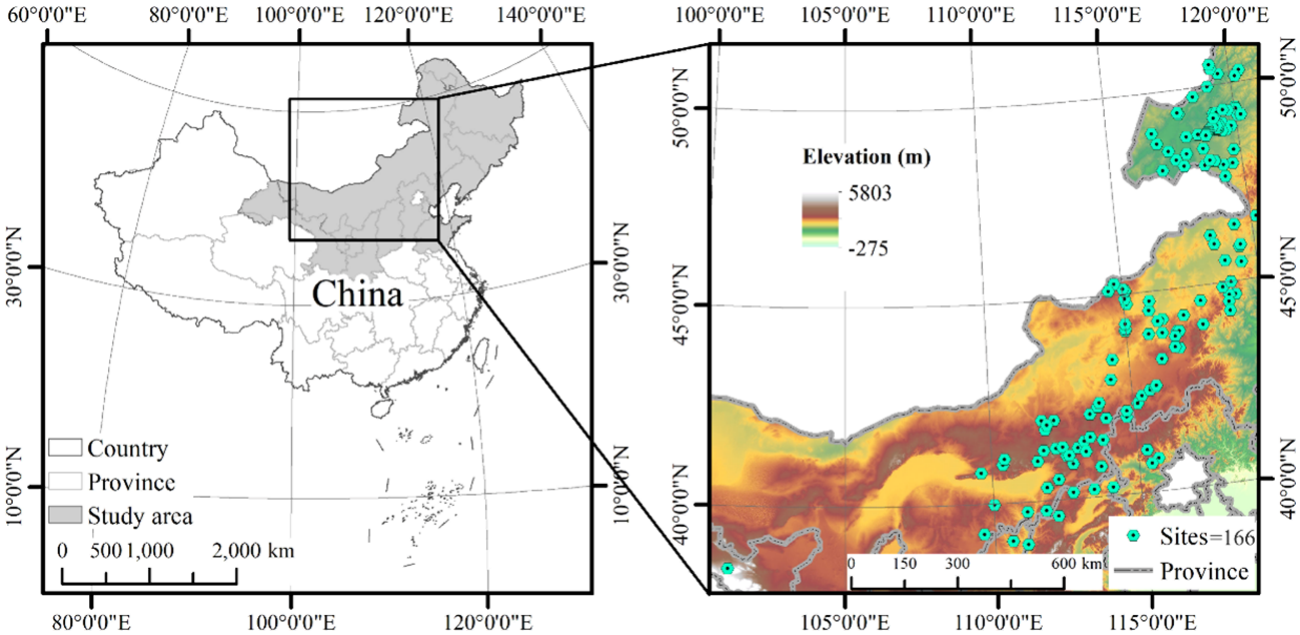
Geographical maps showing the location of the study area in China on the global map, and the sampling sites of *Leymus chinensis* communities within China.

This study was conducted in northern China, with longitudes ranging from 101°14′–125°27′ and latitudes ranging from 38°42′–50°53′’(**Figure 1**). The terrain displays an altitudinal gradient spanning from 129 m to 2085 m, characterized by elevated terrain in the western regions and lower elevations in the eastern zones. The climatic conditions in the area are classified as temperate continental, featuring a mean annual temperature spanning from -3.2°C to 8.1°C. The mean annual precipitation varies from 204 to 501 mm/yr creating a notable rainfall gradient across the study area, with the majority of rainfall occurring during the growing season. The soil types in the study area mainly include chestnut, chernozem, and loess soil. The vegetation types mainly include meadow, meadow steppe, and typical steppe.

Grassland sites dominated by *L. chinensis* were selected as sampling sites. Our vegetation assessments were conducted annually from mid-July to late August between the years 2016 and 2020. This period was chosen to coincide with the peak productivity phase of the grassland ecosystem. Within each site, three randomly established plots, each measuring 1×1 m, were used for data collection; i.e., 498 plots across 166 sites. The following parameters were recorded within each plot: plant height, density, coverage, and composition of the species.

### Data and statistical analyses

#### Computation of patterns in taxonomic and phylogenetic β-diversity

For the computation of taxonomic and phylogenetic *β*-diversity and their two components (i.e., *β*-turnover and *β*-nestedness), we used the pairwise dissimilarity approach between all possible pairs of sites (Baselga, 2013). Based on the presence or absence of species within each site, Jaccard *β*-diversity indices including turnover and nestedness for both taxonomic and phylogenetic aspects were calculated using the “betapart” package (Baselga and Orme, 2012). Species occurring in the sample site were classified into species, genera and families according to the APG IV system. The phylogenetic tree analysis was performed using V.PhyloMaker in R (**Supplementary Figure S1**) (Jin and Qian, 2019), and then, the phylogenetic *β*-diversity indices were calculated. Using the simple bar chart analysis, we showed the patterns of *β*-diversity components.

#### Spatial–environmental drivers of taxonomic and phylogenetic β-diversity

For each sampling site, the longitude, latitude, altitude and slope were determined using the Global Positioning System, compass and ArcGIS. Using the longitude and latitude information of each site, we derived the following environmental factors, i.e., mean annual precipitation (MAP), Mean annual temperature (MAT), precipitation of the coldest quarter (PCQ), minimum temperature of the coldest month (MTCM) and Aridity index (AI) from the WorldClim Database version 2.0 (Fick and Hijmans, 2017). For top-soil nutrients and other related factors such as soil total phosphorus (TP), total potassium (TK), total nitrogen (TN), pH value (H_2_O), available phosphorus (AP), soil exchangeable Mg^2+^ (MG), and exchangeable Ca^2+^ (CA), the data were extracted from the China Dataset of Soil Properties (Shangguan et al., 2013). After that, Mantel correlations were examined to assess the associations of taxonomic and phylogenetic *β*-diversity with spatial and environmental factors. More importantly, variations in each of the taxonomic and phylogenetic *β*-diversity were examined in association with each of the spatial and environmental factors, and their joint effects. The sole and joint effects of spatial and environmental variables on taxonomic and phylogenetic *β*-diversity were determined using the multiple regression model (MRM) (Lichstein, 2007). The residual variance represents the variance described by [1 – (separate and shared variance for environmental and spatial factors)]. The “vegan” package for the Mantel correlation test and the “ecodist” package for the MRM analysis were used (Goslee and Urban, 2007). All data and statistical analyses were done using R software (version 4.2.3). A summary of variables is presented in **Supplementary Table S1**.

## Results

### Patterns of taxonomic and phylogenetic *β*-diversity

**Figure 2:**
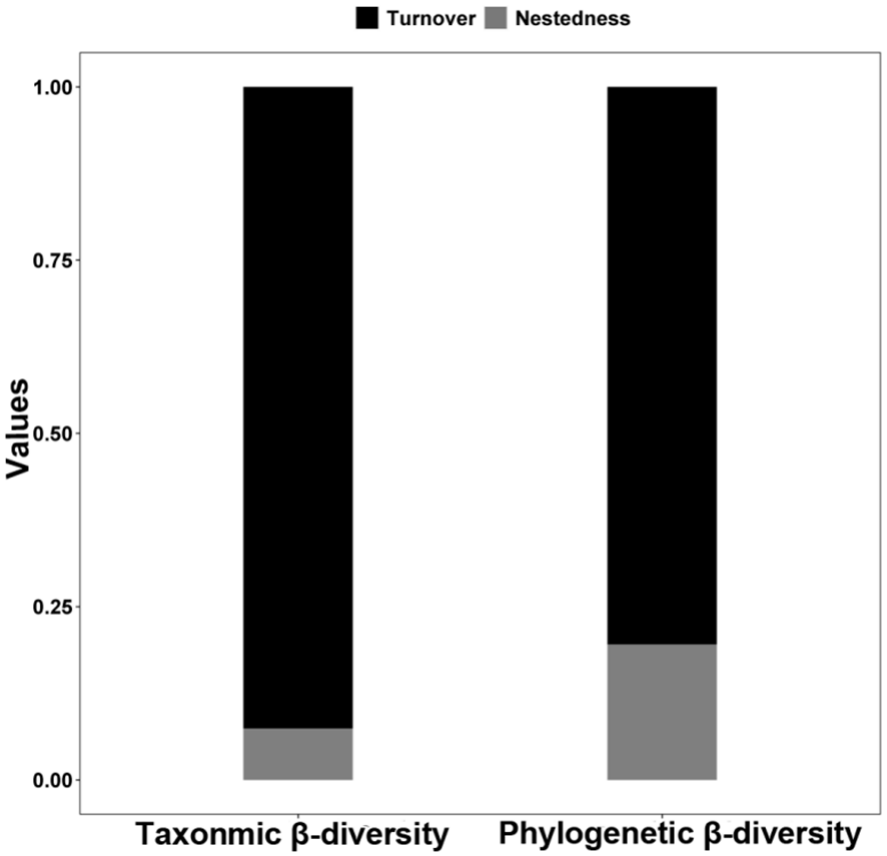
Patterns in the components (turnover and nestedness) of taxonomic and phylogenetic *β*-diversity of *Leymus chinensis* communities in large-scale Chinese grasslands.

The average taxonomic *β*-diversity for the studied *L. chinensis* communities was 0.87, with a *β*-turnover component of 0.81 and a *β*-nestedness component of 0.06. The average phylogenetic *β*-diversity was 0.68, comprising a *β*-turnover component of 0.55 and a *β*-nestedness component of 0.13 (**Figure 2**). The *β*-turnover component exhibited a predominant influence, constituting 92.6% of the taxonomic *β*-diversity and 80.4% of the phylogenetic *β*-diversity. Pearson’s correlation matrix between indices is presented in **Supplementary Figure S2**.

### Associations of spatial and environmental factors with taxonomic and phylogenetic *β*-diversity

**Figure 3:**
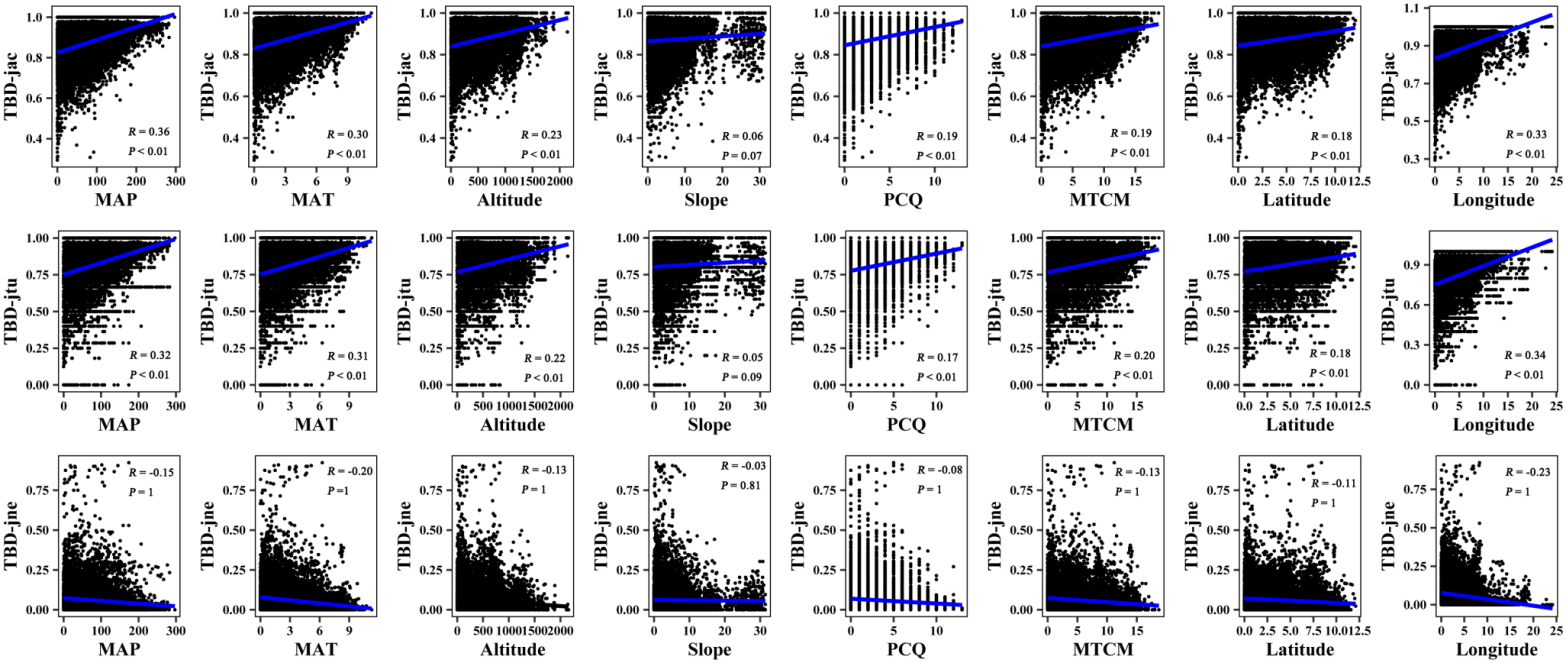
Mantel correlation between taxonomic *β*-diversity and spatial–climate environmental factors of large-scale *Leymus chinensis* grassland communities in China. TBD-jac is taxonomic *β*-diversity (overall); TBD-jtu is taxonomic *β*-turnover; TBD-jne is *β*-nestedness; MAP is mean annual precipitation; MAT is mean annual temperature; PCQ is the precipitation of the coldest quarter; MTCM is the minimum temperature of the coldest month.

**Figure 4:**
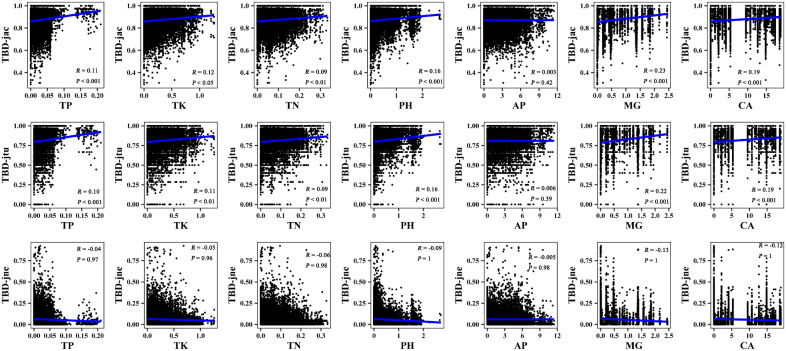
Mantel correlation between taxonomic *β*-diversity and soil factors of large-scale *Leymus chinensis* grassland communities in China. TBD-jac is taxonomic *β*-diversity (overall); TBD-jtu is taxonomic *β*-turnover; TBD-jne is *β*-nestedness; TP is soil total phosphorus; TK is soil total potassium; TN is soil total nitrogen; PH is soil pH value (H_2_O); AP is soil available phosphorus; MG is the soil exchangeable Mg^2+^; CA is soil exchangeable Ca^2+^.

**Figure 5:**
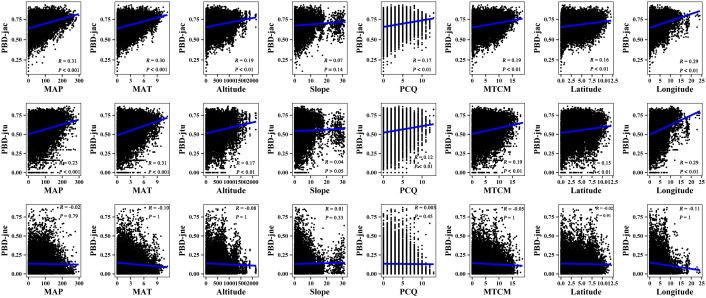
Mantel correlation between phylogenetic *β*-diversity and spatial–climate factors of large-scale *Leymus chinensis* grassland communities in China. TBD-jac is taxonomic *β*-diversity (overall); TBD-jtu is taxonomic *β*-turnover; TBD-jne is *β*-nestedness; MAP is mean annual precipitation; MAT is mean annual temperature; PCQ is the precipitation of the coldest quarter; MTCM is the minimum temperature of the coldest month.

**Figure 6:**
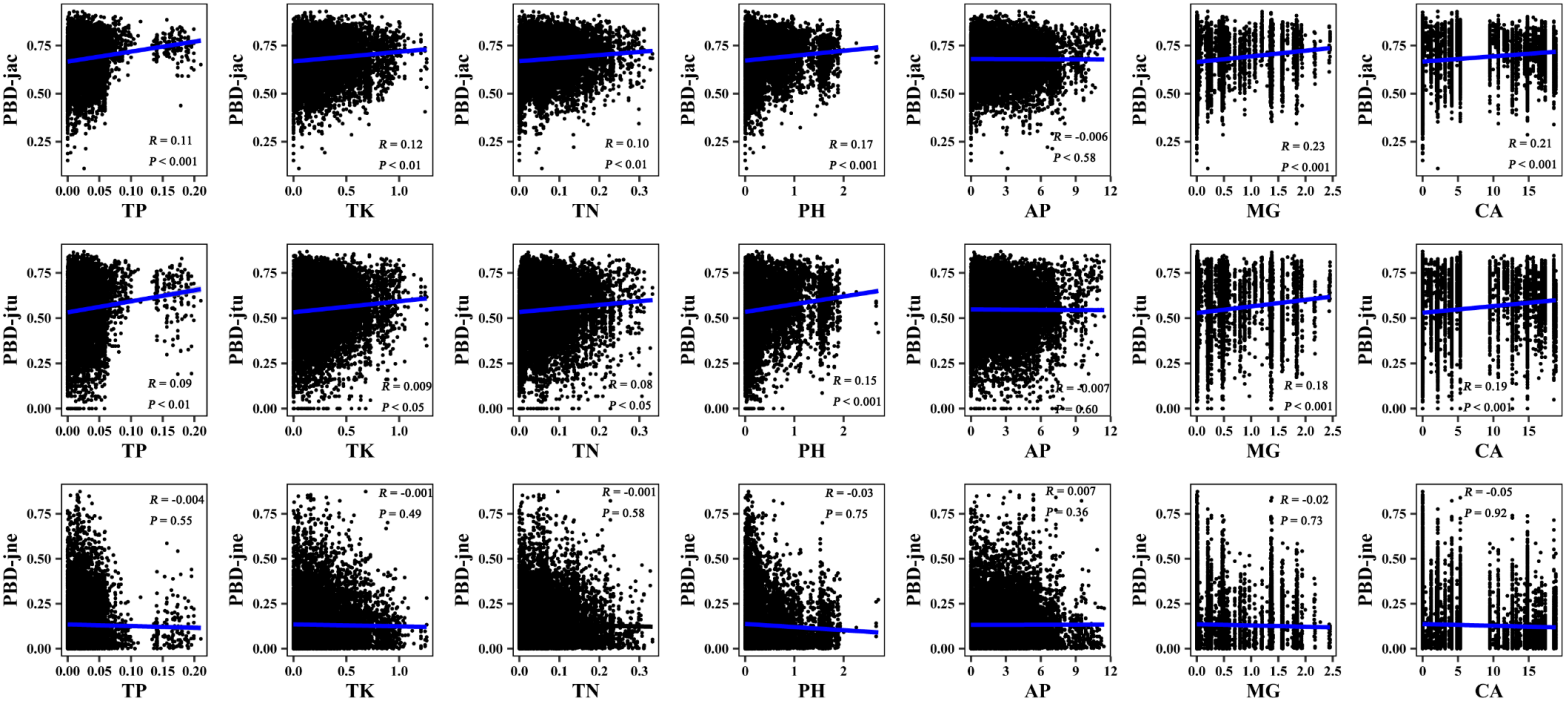
Mantel correlation between phylogenetic *β*-diversity and soil factors of large-scale *Leymus chinensis* grassland communities in China. TBD-jac is taxonomic *β*-diversity (overall); TBD-jtu is taxonomic *β*-turnover; TBD-jne is *β*-nestedness; TP is soil total phosphorus; TK is soil total potassium; TN is soil total nitrogen; PH is soil pH value (H_2_O); AP is soil available phosphorus; MG is the soil exchangeable Mg^2+^; CA is soil exchangeable Ca^2+^.

The results of the Mantel correlation test showed that all spatial–environmental variables were significantly positively correlated with the overall taxonomic and phylogenetic *β*-diversity (**Figures 3**–**6**). The taxonomic and phylogenetic *β*-turnover indices were significantly positively correlated with all spatial–environmental factors, except slope and soil AP, which had no significant correlation. In contrast, the taxonomic and phylogenetic *β*-nestedness indices were not significantly correlated with all spatial–environmental factors (**Figures 3**–**6**). It is worth noting that spatial–climatic factors such as longitude, MAP and MAT were the strongest predictors of both taxonomic and phylogenetic *β*-diversity and *β*-turnover, followed by other spatial–environmental factors (**Figures 3**–**6**). Pearson’s correlation matrix between spatial–and environmental factors is presented in **Supplementary Figure S3**.

### Relative importance of spatial and environmental factors with taxonomic and phylogenetic *β*-diversity

**Figure 7:**
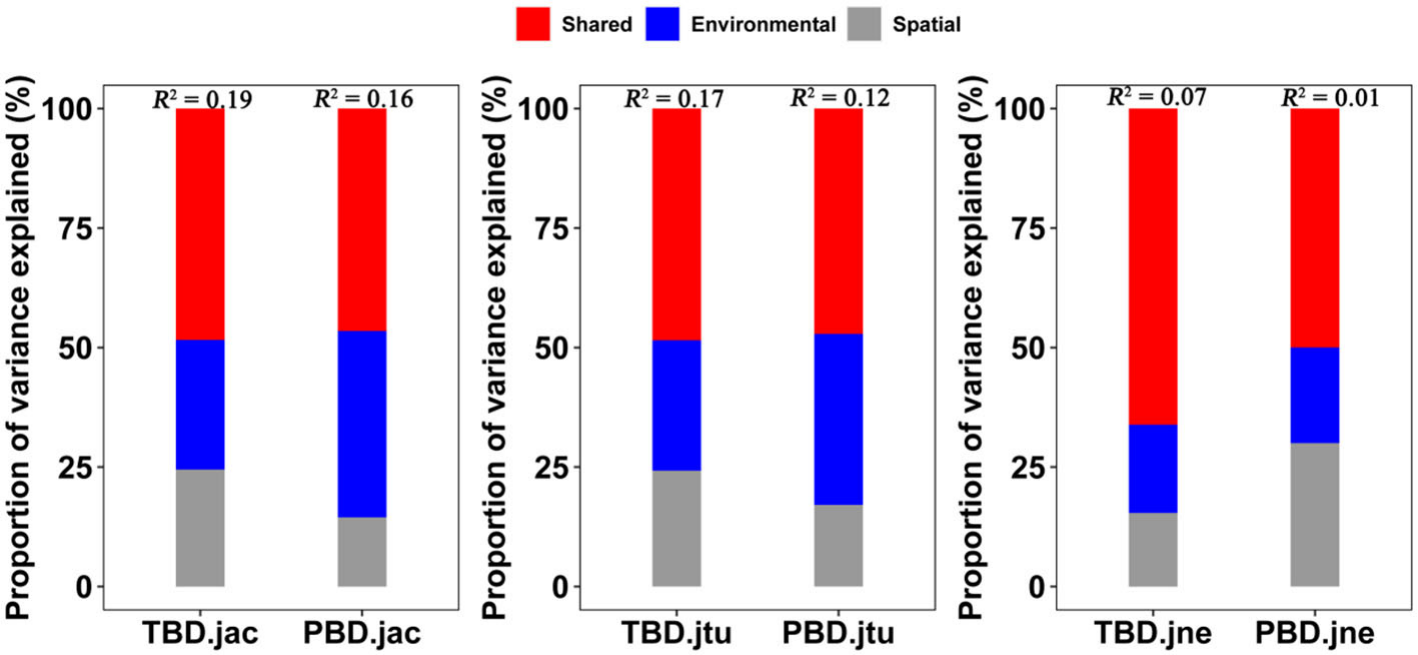
Interpretation rates of variance partitioning of taxonomic and phylogenetic *β*-diversity in large-scale *Leymus chinensis* grassland communities in China. TBD-jac is taxonomic *β*-diversity (overall); TBD-jtu is taxonomic *β*-turnover; TBD-jne is *β*-nestedness.

The variance partitioning analysis, based on the MRM, showed that the combined effects of spatial and environmental factors accounted for 19% and 16% of the variation in the taxonomic and phylogenetic *β*-diversity (overall), 17% and 12% of the variation in the *β*-turnover, and 7% and 1% of the variation in the *β*-nestedness, respectively (**Figure 7**). Specifically, the independent effects of environmental factors explained greater variation than the independent effects of spatial factors (i.e., < 2%), accounting for 5.4% and 6.2% of the variation in the taxonomic and phylogenetic *β*-diversity (overall), 4.5% and 4.4% of the variation in the *β*-turnover, and 1.2% and 0.2% of the variation in the *β*-nestedness component, respectively. These findings suggest that while both spatial and environmental somehow contribute to explaining *β*-diversity, their shared role has a greater effect than their single role (**Figure 7**; **Supplementary Figure S4**).

## Discussion

In this study, we comprehensively analyzed the patterns and spatial–environmental drivers of taxonomic and phylogenetic *β*-diversity and their two components (*β*-turnover and *β*-nestedness) within the widely distributed *L. chinensis* communities in northern China. The results indicate that *β*-turnover contributed substantially to the *β*-diversity (overall) than *β*-nestedness, regardless of taxonomic or phylogenetic identification of plant species. Interestingly, taxonomic and phylogenetic *β*-diversity (overall) and *β*-turnover, but not *β*-nestedness, were significantly positively affected by most of the spatial and environmental variables. Moreover, although environmental factors explained more variation in *β*-diversity than spatial factors, their joint effects were higher in explaining patterns in large-scale *L. chinensis* grassland communities in northern China.

Our results show that the composition patterns of taxonomic and phylogenetic *β*-diversity (overall) of *L. chinensis* communities in China exhibited uniformity, with both primarily driven by the *β*-turnover component. At larger spatial scales, the turnover component of *β*-diversity may be associated with the species complementarity effect, but dominant species, or functionally important species, can actually vary among communities due to the species selection effect (Mori et al., 2018). This aligns with the findings of numerous previous studies conducted in different ecosystems, such as grasslands and forests, indicating that alterations in species composition within communities are predominantly influenced by species substitution (Yakimov et al., 2020). The distribution of our study sites, primarily located on the Mongolian Plateau, likely contributed to the observed consistent patterns probably because the Mongolian Plateau experienced less impact from the Last Glacial Maximum, allowing for increased species survival. Furthermore, the region has exhibited significant warming and wetting trends since the Last Glacial Maximum, facilitating more species survival and leading to higher species turnover in the area (Zheng et al., 2019). As we investigated large-scale *L. chinensis*-dominated communities distributed in different habitats, the structural composition and origin of the communities tended to be similar due to their shared developmental history. This shared evolutionary history leads to the coexistence of closely related species within the same region, typically leading to lower phylogenetic *β*-diversity when compared to taxonomic *β*-diversity (Cavender-Bares et al., 2009; Mori et al., 2018). A comparable phenomenon has been observed in the assessment of *β*-diversity among angiosperms in studies conducted in other regions (Qin et al., 2019; Qian et al., 2020).

In comparison to spatial factors, we found that the role of environmental (including both climate and soil) factors was more pronounced in shaping taxonomic and phylogenetic *β*-diversity (and more importantly *β*-turnover) of *L. chinensis* grassland communities. This result can be attributed to the fact the impacts of environmental factors outweigh the effects of spatial factors, underscoring the pivotal role of environmental factors in driving changes in plant community species composition and ecosystem functioning (Page and Shanker, 2018; Yi et al., 2020; van der Plas et al., 2023). Specifically, mean annual precipitation and temperature were identified as the predominant factors exerting significant influences on both taxonomic and phylogenetic *β*-diversity of *L. chinensis* communities, indicating that climate change may largely influence the species diversity, structure and functioning of studied plant communities (Pugnaire et al., 2019; Berdugo et al., 2020; Yao et al., 2022). However, the topographic slope as compared to elevation exhibited no substantial effect on taxonomic and phylogenetic *β*-diversity, probably linked to the topographic-centric distribution of *L. chinensis* communities that predominantly occurs in the lower parts of the lower hills due to favourable climatic and soil conditions. In these areas, the community-building species *L. chinensis* occupies more ecological niches, resulting in a simpler composition of other species in the community, thereby allowing the survival of closely related species, which leads to its significant association with phylogenetic *β*-diversity (Satdichanh et al., 2019). We also found that most of the soil factors, except soil AP, were significantly correlated with taxonomic and phylogenetic *β*-diversity, indicating the role of soil fertility effect on plant growth through niche complementarity mechanisms (Quesada et al., 2012). Nonetheless, it is also debated that climatic factors outweigh soil factors in their contribution to *β*-diversity, with climate generally regulating community assembly at larger scales (Qian et al., 2013), whereas soil’s impact becomes more pronounced at smaller scales (Zhang et al., 2011).

The sexuality of *L. chinensis* grassland community is mainly influenced by environmental filtering that primarily comprises perennial rhizomatous grasses, thereby predominantly relying on asexual reproduction, resulting in minimal seed reproduction probability (Ott and Hartnett, 2011; Russell et al., 2017). As a result, the *β*-diversity of *L. chinensis* community is primarily governed by environmental factors, consistent with the findings of other studies conducted in grassland areas (Li et al., 2021). Notably, both taxonomic and phylogenetic *β*-diversity of *L. chinensis* communities were more explained by combined spatial and environmental factors than by environment or space alone, indicating the context-dependency effect (Catford et al., 2021). Through variance partitioning analysis, we show that a considerable portion of the *β*-diversity of *L. chinensis* community remains unexplained, which could be attributed, at least in part, to the absence of analysis on functional traits, such as seed characteristics that indicate dispersal abilities (Sreenivasulu and Wobus, 2013). Additionally, the limited scope of our spatial–environmental conditions, which only considered a small number of factors such as climate, topography, and soils, might have resulted in the overlooking of the influence of other variables, including various soil trace elements and water content (Myers et al., 2000; Zhang et al., 2020). Also, some studies have shown that factors such as paleoclimate and human activities are important factors influencing *β*-diversity (Dobrovolski et al., 2012; Qin et al., 2019). Therefore, to achieve a comprehensive understanding of the underlying mechanisms driving *β*-diversity formation within *L. chinensis* communities, future research efforts should encompass the integration of more environmental variables, functional characteristics, and human disturbances.

## Conclusions

We show that *β*-turnover plays a significant role in the overall taxonomic and phylogenetic *β*-diversity, surpassing the contribution of *β*-nestedness. Intriguingly, both taxonomic and phylogenetic *β*-diversity, as well as *β*-turnover rather than *β*-nestedness, are strongly influenced by various spatial and environmental factors such as favorable climatic and soil fertility conditions. Furthermore, while environmental factors explain a greater proportion of the variation in *β*-diversity, the combined effects of spatial and environmental factors are particularly important in understanding the patterns observed in the large-scale *L. chinensis* grassland communities in northern China. We contend that the *β*-turnover component holds significant control in shaping the processes of plant community formation, thereby influencing the functioning and services provided by grasslands. By incorporating *β*-diversity into grassland management strategies, we can enhance the provision of vital ecosystem services that bolster human welfare, serving as a resilient barrier against the adverse effects of climate change at regional and global scales. Regrettably, prevailing management policies frequently overlook *β*-diversity as a viable metric for assessing biodiversity, owing to the limited connection between scientific findings, policy formulation, and implementation.

## Data availability statement

The original contributions presented in the study are included in the article/**Supplementary Material**. Further inquiries can be directed to the corresponding authors.

## Author contributions

ZY: Writing – review & editing, Writing – original draft, Investigation, Data curation, Conceptualization. YX: Writing – review & editing, Investigation. ZM: Writing – review & editing, Data curation. LZ: Writing – original draft, Investigation, Writing – review & editing, Data curation. WM: Writing – review & editing, Investigation. JG: Funding acquisition, Writing – review & editing. AA: Data curation, Writing – review & editing.
