# Supplementary material for: Plant beta-turnover rather than nestedness shapes overall taxonomic and phylogenetic beta-diversity triggered by favorable spatial–environmental conditions in large-scale Chinese grasslands
Source: Front Plant Sci. 2024 May 28;15:1285787. doi: 10.3389/fpls.2024.1285787 (PMC11187821; doi:10.3389/fpls.2024.1285787)
Supplement: Supplementary file 1 [file DataSheet_1.doc]

**Plant beta-turnover rather than nestedness shapes overall taxonomic and phylogenetic beta-diversity triggered by favorable spatial–environmental conditions in large-scale Chinese grasslands**

**Zhenyu Yao^1,2^, Yue Xin^3^, Zhaoxia Ma^4^, Jianying Guo^1,2^, Wenkui Mu^5^, Liqing Zhao^3*^, Arshad Ali^6*^**

^1^Yinshanbeilu Grassland Eco-hydrology National Observation and Research Station, China Institute of Water Resources and Hydropower Research, Beijing 100038, PR China

^2^Institute of Water Resources for Pastoral Areas, Ministry of Water Resources, Hohhot, 010020.China

^3^Inner Mongolia Key Laboratory of Grassland Ecology and School of Ecology and Environment, Inner Mongolia University, Hohhot, 010021, China

^4^ Beijiao Park, Hohhot, 010051, China

^5^Inner Mongolia Hohhot Meteorological Bureau, Hohhot, 010020, China

^6^Forest Ecology Research Group, College of Life Sciences, Hebei University, Baoding, 071002, Hebei, China

*Corresponding authors

**Liqing Zhao**

Inner Mongolia University, Hohhot, 010021, China.

Email address: zhaotieniu@126.com

**Arshad Ali**

Forest Ecology Research Group, College of Life Sciences, Hebei University, Baoding, 071002, Hebei, China

Email addresses: arshadforester@gmail.com / arshadforester@hbu.edu.cn

ORCID: 0000-0001-9966-2917

**Table S1.** Descriptive statistics of the variables across 166 meta-sites (i.e., the mean value of 3 plots per site) of grasslands dominated by *Leymus chinensis* in northern China.

| Variable | Unit | Mean | S.D. | Min. | Max. |
| --- | --- | --- | --- | --- | --- |
| ***Pooled data (n = 166)*** |  |  |  |  |  |
| Taxonomic β-diversity (TBD-jac) | / | 0.87 | 0.09 | 0.29 | 1 |
| Taxonomic β-turnover component (TBD-jtu) | / | 0.81 | 0.14 | 0 | 1 |
| Taxonomic β-nestedness component (TBD-jne) | / | 0.06 | 0.08 | 0 | 0.92 |
| Phylogenetic β-diversity (PBD-jac) | / | 0.68 | 0.10 | 0.11 | 0.93 |
| Phylogenetic β-turnover component (PBD-jtu) | / | 0.55 | 0.15 | 0 | 0.87 |
| Phylogenetic β-nestedness component (PBD-jne) | / | 0.13 | 0.12 | 0 | 0.87 |
| Latitude | Decimal degrees | 44.98 | 3.30 | 38.43 | 50.53 |
| Longitude | Decimal degrees | 116.60 | 3.63 | 101.14 | 125.28 |
| Altitude | m | 1133.48 | 423.56 | 129.40 | 2285.00 |
| Slope | ° | 4.98 | 4.57 | 0.35 | 31.76 |
| Mean annual precipitation (MAP) | mm | 325.14 | 62.66 | 204.00 | 501.00 |
| Mean annual temperature (MAT) | °C | 1.50 | 2.51 | -3.22 | 8.06 |
| precipitation of the coldest quarter (PCQ) | mm | 8.46 | 2.55 | 4.00 | 17.00 |
| minimum temperature of the coldest month (MTCM) | °C | -25.63 | 4.55 | -34.6 | -15.90 |
| Total phosphorus (TP) | g/100g | 0.07 | 0.02 | 0.02 | 0.23 |
| Total potassium (TK) | g/100g | 2.06 | 0.05 | 1.55 | 2.81 |
| pH value (H_2_O) (PH) | / | 8.13 | 0.36 | 6.57 | 9.28 |
| Total nitrogen (TN) | g/100g | 0.14 | 0.06 | 0.01 | 0.34 |
| Available phosphorus (AP) | mg/kg | 4.89 | 2.43 | 1.48 | 12.92 |
| Exchangeable Ca^2+^ (CA) | me/100 g | 15.92 | 5.47 | 0.19 | 19.02 |
| Exchangeable Mg^2+^ (MG) | me/100 g | 1.60 | 0.57 | 0.35 | 2.80 |


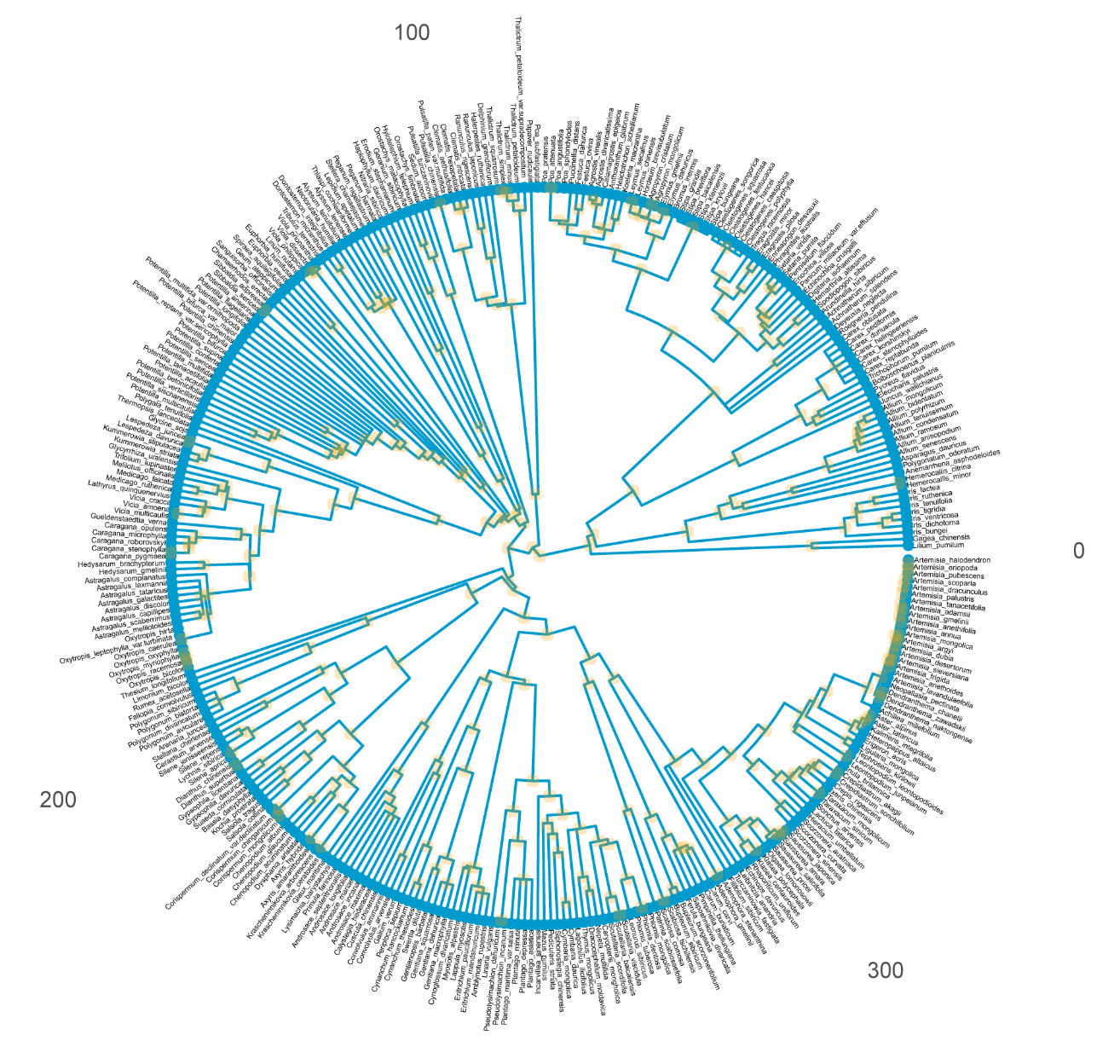


**Figure S1** Phylogenetic tree of *Leymus chinensis* community


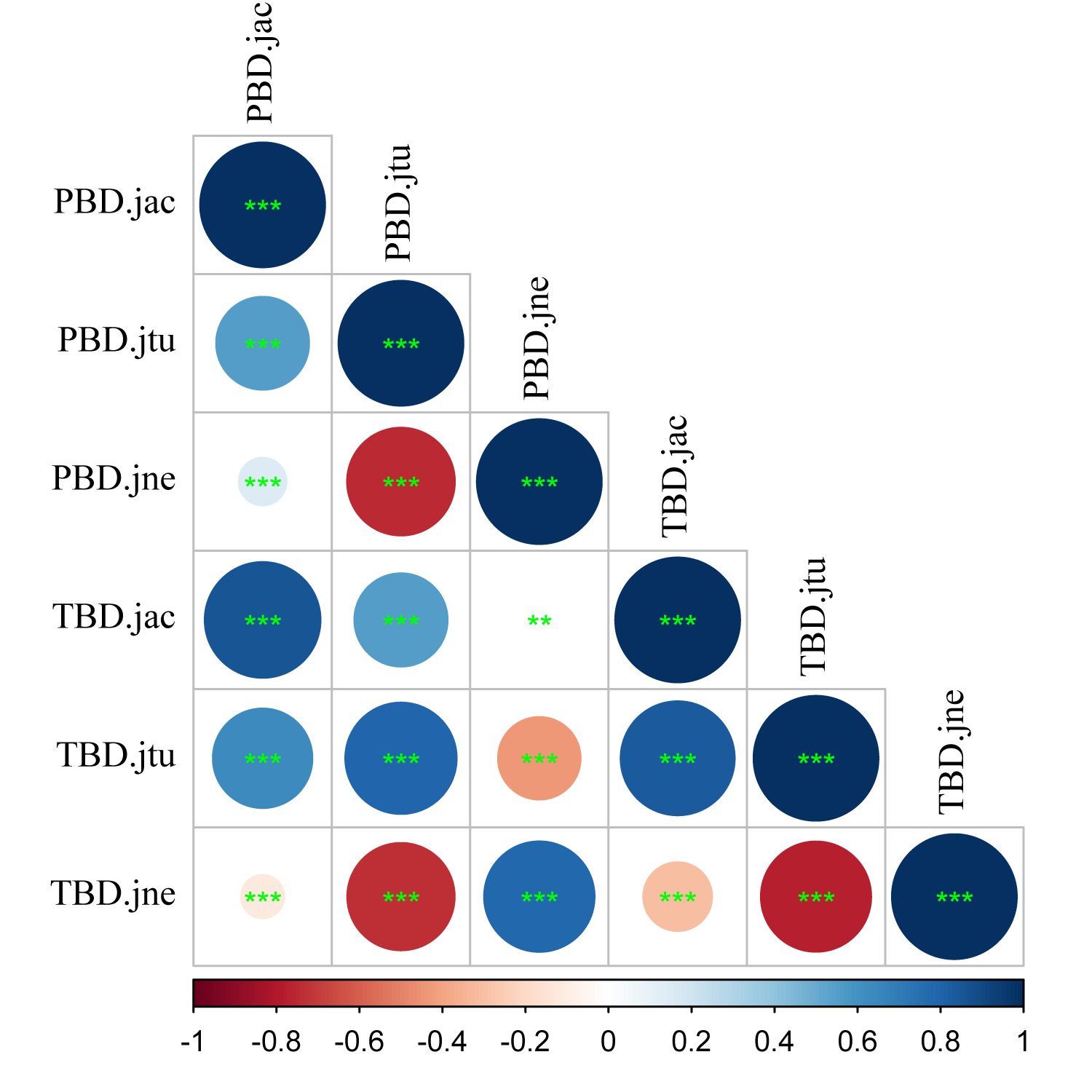


**Figure S2** The Pearson’s correlation coefficients matrix for tested variables for linking beta indices of *Leymus chinensis* communities in northern China. Dark red to dark blue colors represent negative to positive correlations, with ****, P < 0.001; **, P < 0.01; *, P < 0.05. See Table S1 for abbreviations.


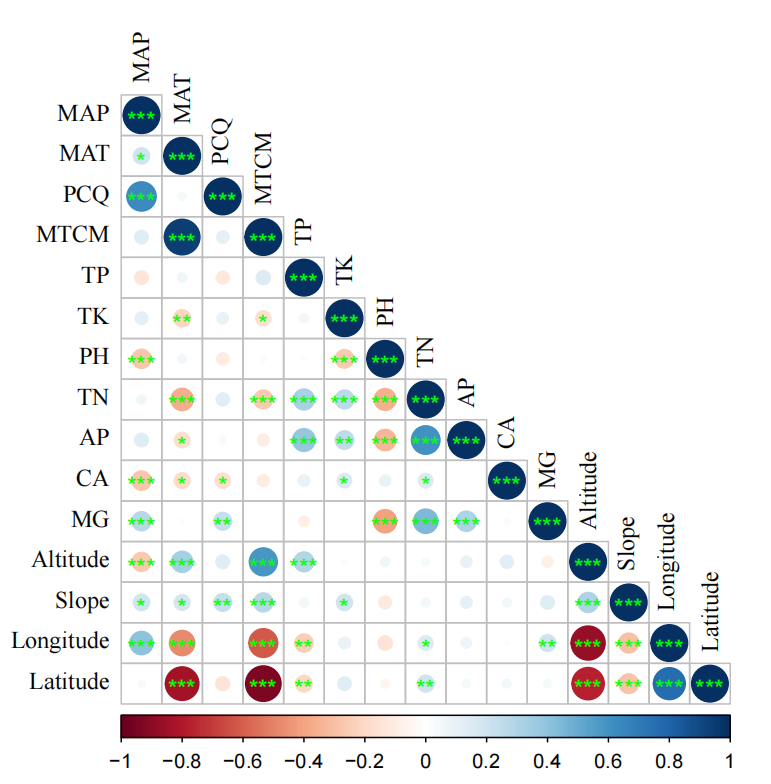


**Figure S3** The Pearson’s correlation coefficients matrix for tested variables for linking climatic factors, topographic, soil, longitude and latitude of *Leymus chinensis* communities in northern China. Dark red to dark blue colors represent negative to positive correlations, with ****, P < 0.001; **, P < 0.01; *, P < 0.05. See Table S1 for abbreviations.


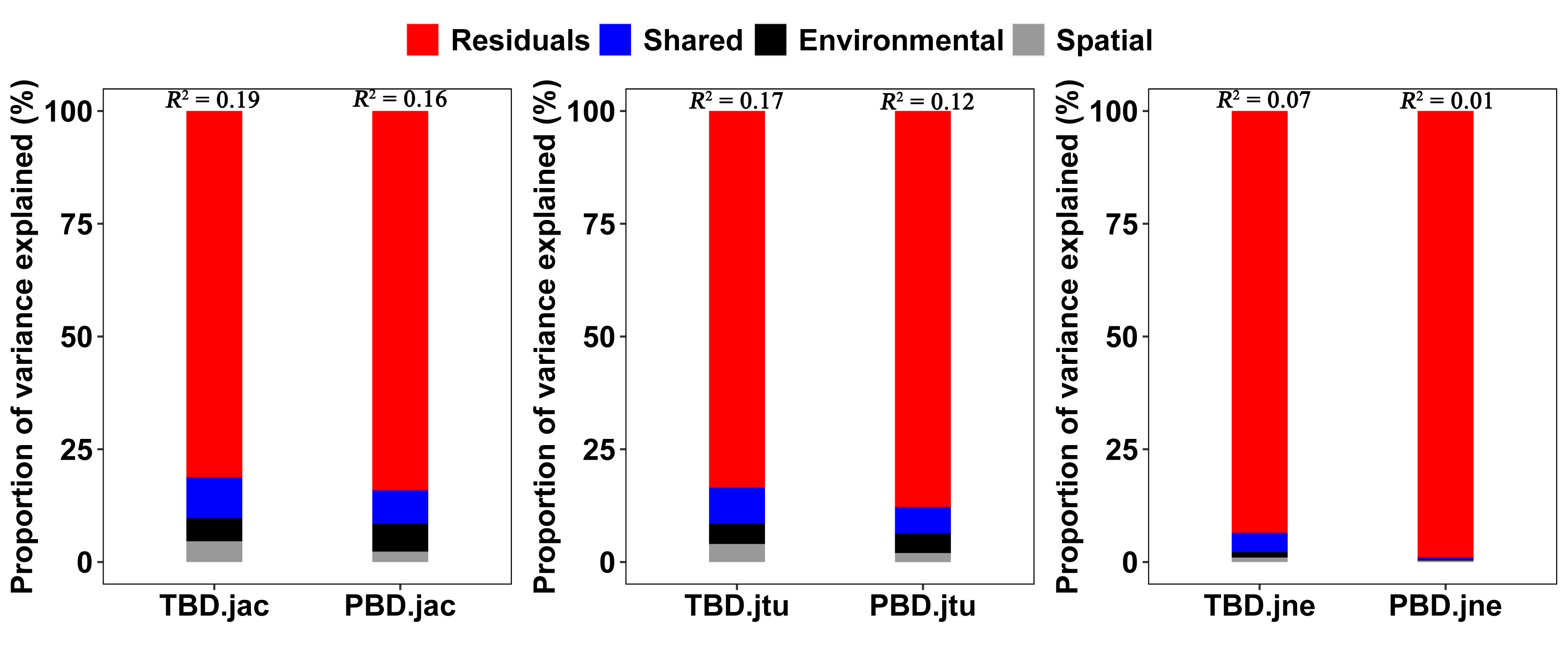


**Figure S4** Variance partitioning of taxonomic and phylogenetic *β*-diversity in large-scale *Leymus chinensis* grassland communities in China.
